# Mutation analysis of 12 genes in Chinese families with congenital cataracts

**Published:** 2011-08-16

**Authors:** Wenmin Sun, Xueshan Xiao, Shiqiang Li, Xiangming Guo, Qingjiong Zhang

**Affiliations:** State Key Laboratory of Ophthalmology, Zhongshan Ophthalmic Center, Sun Yat-sen University, Guangzhou, China

## Abstract

**Purpose:**

To identify mutations in 12 genes in Chinese families with congenital cataracts.

**Methods:**

Twenty five families with congenital cataracts involved in this study. The coding exons and adjacent intronic regions of 12 genes were analyzed by cycle sequencing, including the alpha A crystallin (*CRYAA*), alpha B crystallin (*CRYAB*), beta A1 crystallin (*CRYBA1*), beta A4 crystallin (*CRYBA4*), beta B1 crystallin (*CRYBB1*), beta B2 crystallin (*CRYBB2*), beta B3 crystallin (*CRYBB3*), gamma C crystallin (*CRYGC*), gamma D crystallin (*CRYGD*), gamma S crystallin (*CRYGS*), alpha 3 gap junction protein (*GJA3*), and alpha 8 gap junction protein (*GJA8*) genes. Novel variants were further evaluated in 96 normal controls.

**Results:**

Nine mutations were identified in 10 of the 25 families (40%), including 5 novel (c.350_352delGCT in *CRYAA*, c.205C>T in *CRYAB*, c.106G>C in *CRYGD*, c.77A>G in *CRYGS*, c.1143_1165del23 in *GJA3*) and 4 known (c.292G>A in *CRYAA*; c.215+1G>A and c.272_274delGAG in *CRYBA1*, and c.176C>T in *GJA3*). All novel mutations were predicted to be pathogenic and were not present in 96 controls.

**Conclusions:**

Mutations in the 12 genes encoding crystallins and connexins were responsible for 40% Chinese families with congenital cataracts. Our results enriched our knowledge on the molecular basis of congenital cataracts in Chinese population.

## Introduction

Congenital cataract is a leading cause of childhood blindness, accounting for 10~38% of blindness in children. The prevalence of congenital cataract is estimated to be 0.6~6 per 10,000 live births [1,2]. Various etiological factors have been reported, including infection, neonatal asphyxia, malnutrition, and genetic defects. It was reported that 8.3%~25% of congenital cataracts were inherited [3]. Several genes have been identified to be associated with congenital cataracts [3,4], such as genes encoding crystallins, connexins and other membrane proteins, beaded filament proteins, growth and transcription factors, and others.

For cataract families with identified mutations, it has been suggested that about half had mutations in crystallins and a quarter in connexins (gap junction proteins) [3]. So far, mutations in 10 crystallin genes and 2 connexin genes have been identified to be responsible for congenital cataracts, including alpha A crystallin (*CRYAA*, OMIM 123580) [5-12], alpha B crystallin (*CRYAB*, OMIM 123590) [10,13-15], beta A1 crystallin (*CRYBA1*, OMIM 123610) [16-23], beta A4 crystallin (*CRYBA4*, OMIM 123631) [24,25], beta B1 crystallin (*CRYBB1*, OMIM 600929) [26-29], beta B2 crystallin (*CRYBB2*, OMIM 123620) [30-34], beta B3 crystallin (*CRYBB3*, OMIM 123630) [35,36], gamma C crystallin (*CRYGC*, OMIM 123680) [12,37-40], gamma D crystallin (*CRYGD*, OMIM 123690) [37,41-43], gamma S crystallin (*CRYGS*, OMIM 123730) [10,44,45], alpha 3 gap junction protein (*GJA3*, OMIM 121015) [34,46-50], and alpha 8 gap junction protein (*GJA8*, OMIM 600897) [40,51-56]. Several mutations in these genes have been identified but most reports are either based on one to a few gene(s) or one to a few family(ies). The exact mutation frequency of these genes in congenital cataract is unclear as comprehensive analysis of all the 12 genes in the same set of families is absent although a few studies reported analysis of some of them [10,29,36].

In this study, molecular genetic analysis was performed on all these 12 genes in 25 Chinese families with congenital cataract. Nine mutations, including 5 novel and 4 known, were identified in 10 of the 25 families (40%).

## Methods

### Patients

Written informed consent conforming to the tenets of the Declaration of Helsinki and following the Guidance of Sample Collection of Human Genetic Diseases (863-plan) by the Ministry of Public Health of China were obtained from the participating individuals or their guardians before the study. Twenty five Chinese families with congenital cataracts were enrolled in this study. Genomic DNA was prepared from leukocytes of peripheral venous blood. This study was approved by the Institutional Review Board of the Zhongshan Ophthalmic Center, Sun Yat-sen University, Guangzhou, China.

### Mutational screening

Bioinformation of the 12 genes was listed in Table 1, which was obtained from the National Center for Biotechnology Information (NCBI). Polymerase chain reaction (PCR) was used to amplify the coding exons and adjacent intronic sequences of the 12 genes. The primer sequences used to amplify each coding exon and its adjacent regions of the 12 genes were referred to the previous publications [36,57-59] with modification for some primers (Appendix 1). The nucleotide sequences of PCR products were determined with the ABI BigDye Terminator cycle sequencing kit v3.1 on a genetic analyzer (ABI Applied Biosystems, Foster City, CA). Variations were identified by importing the sequencing results from patients and consensus sequences from the NCBI human genome database into the SeqManII program of the Lasergene package (DNAStar Inc., Madison, WI). Potential variants detected in patients were further evaluated in 96 normal controls by cycle sequencing.

**Table 1:** Genomic information of the 12 genes referred in this study.

**Gene**	**Genomic DNA**	**mRNA**	**Protein**
*CRYAA*	NC_000021.8	NM_000394.2	NP_000385.1
*CRYAB*	NC_000011.9	NM_001885.1	NP_001876.1
*CRYBA1*	NC_000017.10	NM_005208.4	NP_005199.2
*CRYBA4*	NC_000022.10	NM_001886.2	NP_001877.1
*CRYBB1*	NC_000022.10	NM_001887.3	NP_001878.1
*CRYBB2*	NC_000022.10	NM_000496.2	NP_000487.1
*CRYBB3*	NC_000022.10	NM_004076.3	NP_004067.1
*CRYGC*	NC_000002.11	NM_020989.3	NP_066269.1
*CRYGD*	NC_000002.11	NM_006891.3	NP_008822.2
*CRYGS*	NC_000003.11	NM_017541.2	NP_060011.1
*GJA3*	NC_000013.10	NM_021954.3	NP_068773.2
*GJA8*	NC_000001.10	NM_005267.4	NP_005258.2

The information is based on human genome (Build 37.2).

### Database and online tools

Mutation description followed the recommendation of the Human Genomic Variation Society (HGVS). The effects of novel missense mutations on the encoded proteins were further evaluated by Polymorphism Phenotyping (PolyPhen-2) [60,61] and Sorting Intolerant From Tolerant (SIFT) [62] at the protein level.

## Results

**Figure 1:**
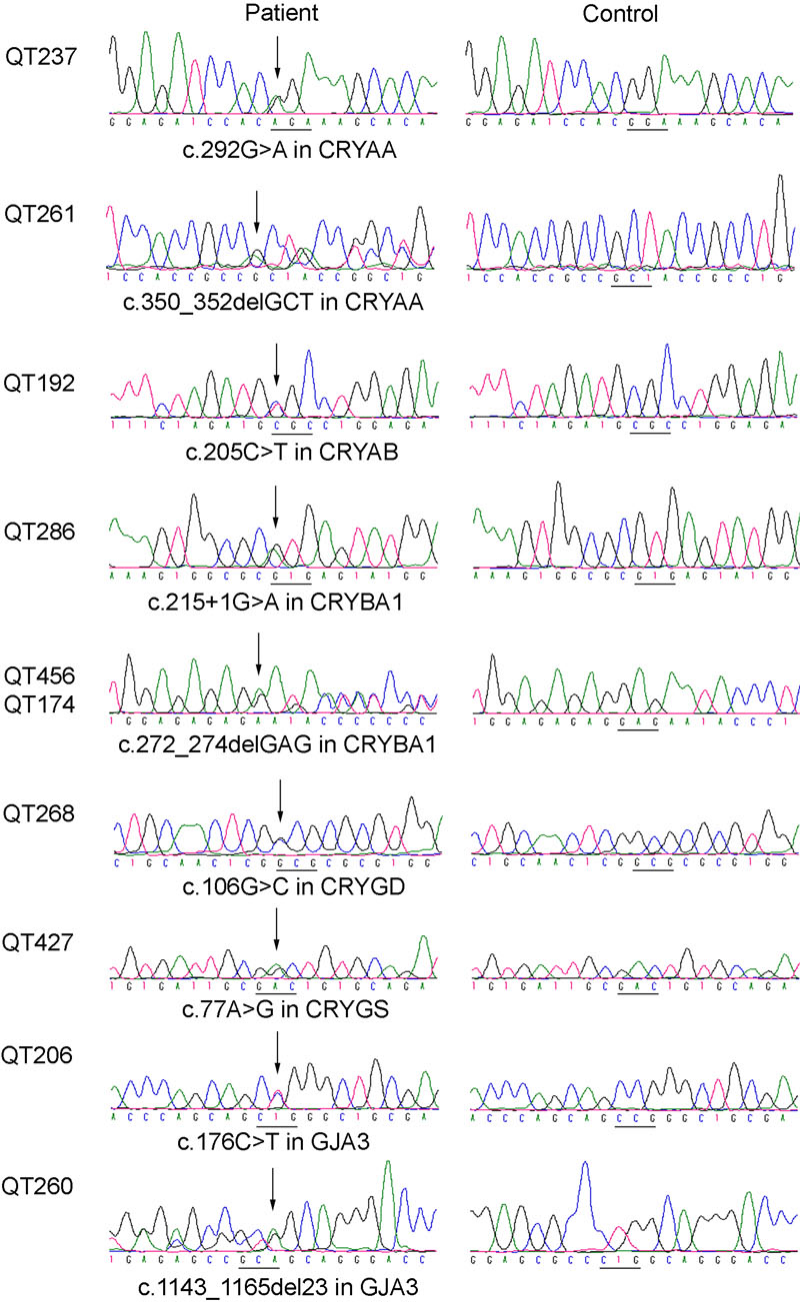
Sequence chromatography. The family number of each proband was shown in the left column. Sequences with mutations from probands were shown in the middle and those from normal controls were aligned on the right column. For families QT456 and QT174, only the mutant sequence of the proband from family QT456 was shown as both probands had the same mutation. Each mutation was described under the corresponding sequence.

**Figure 2:**
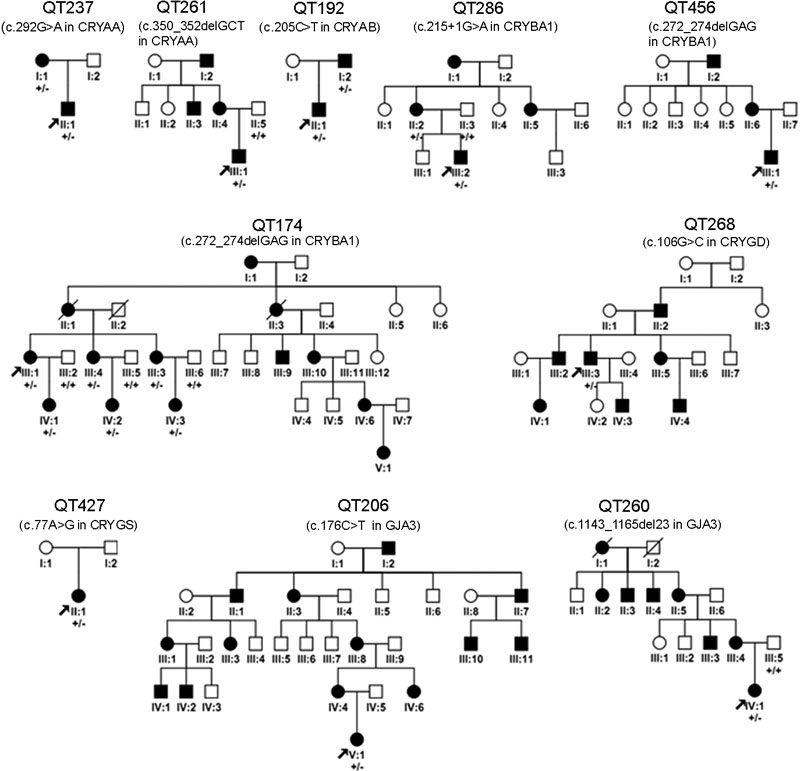
Pedigrees of the ten families with mutations. The family numbers and their corresponding mutations were shown just above the pedigree. The +/− indicated heterozygous mutation and the +/+ indicated wild type.

Based on complete analysis of the coding exons and their adjacent intronic regions in the 12 genes in 25 families, nine heterozygous mutations were detected in 6 genes in 10 families (Table 2, Figure 1). Of the nine mutations, five were novel (c.350_352delGCT in *CRYAA*, c.205C>T in *CRYAB*, c.106G>C in *CRYGD*, c.77A>G in *CRYGS*, c.1143_1165del23 in *GJA3*) and four were known (c.292G>A in *CRYAA*; c.215+1G>A and c.272_274delGAG in *CRYBA1*, and c.176C>T in *GJA3*). The c.272_274delGAG mutation in *CRYBA1* was present in two unrelated families. The pedigrees and cosegregation analyses of the 10 families with identified mutations were shown in Figure 2. All 5 novel mutations were not present in 96 normal controls.

**Table 2:** Summary of mutations detected in patients with congenital cataracts in this study.

**Gene**	**Nucleotide change**	**Amino acid change**	**Effect prediction**		**Frequency in**		**Note**	**References**
** **	** **	** **	**PolyPhen-2**	**SIFT**	**patients**	**controls**	** **	** **
*CRYAA*	c.292G>A	p.Gly98Arg	probably damaging	damaging	1/25	N/A	reported*	[7]
*CRYAA*	c.350_352 delGCT	p.[Arg117His, Tyr118del]	N/A	N/A	1/25	0/96	novel	
*CRYAB*	c.205 C>T	p.Arg69Cys	probably damaging	damaging	1/25	0/96	novel	
*CRYBA1*	c.215+1G>A	splicing donor abolished	N/A	N/A	1/25	N/A	reported	[10,16,19,20,63]
*CRYBA1*	c.272_274 delGAG	p.Gly91del	N/A	N/A	2/25	N/A	reported	[18,21-23]
*CRYGD*	c.106G>C	p.Ala36Pro	benign	damaging	1/25	0/96	novel	
*CRYGS*	c.77 A>G	p.Asp26Gly	probably damaging	damaging	1/25	0/96	novel	
*GJA3*	c.1143_1165del23	p.381fs*48	N/A	N/A	1/25	0/96	novel	
*GJA3*	c.176 C>T	p.Pro59Leu	probably damaging	damaging	1/25	N/A	reported	[36,50]

*Reported indicates that these known mutations have been reported previously in other families.

**Figure 3:**
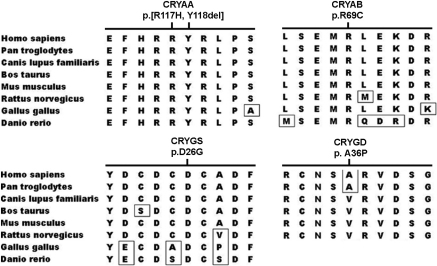
Conservation alignments of protein orthologs for 4 of the 5 novel mutations. The regions with p.[R117H,Y118del] in *CRYAA*, p.R69C in *CRYAB*, and p.D26G in *CRYGS* are highly conserved, while the p.A36P in *CRYGD* is not conserved (only 6 of the 8 orthologs available for *CRYGD*).

Three of the five novel variants were missense mutations. Of the three, the c.205C>T (p.Arg69Cys) in *CRYAB* and the c.77A>G (p.Asp26Gly) in *CRYGS* involved highly conserved residues while the other one (p.Ala36Pro in *CRYGD*) replaced a nonconserved residue (Figure 3). The novel c.350_352delGCT mutation in *CRYAA* resulted in substitution of arginine at position 117 and deletion of tyrosine at position 118, where the two residues are highly conserved (Figure 3). The c.1143_1165del23 mutation resulted in frameshift with additional 48 new residues from residue 381.

**Figure 4:**
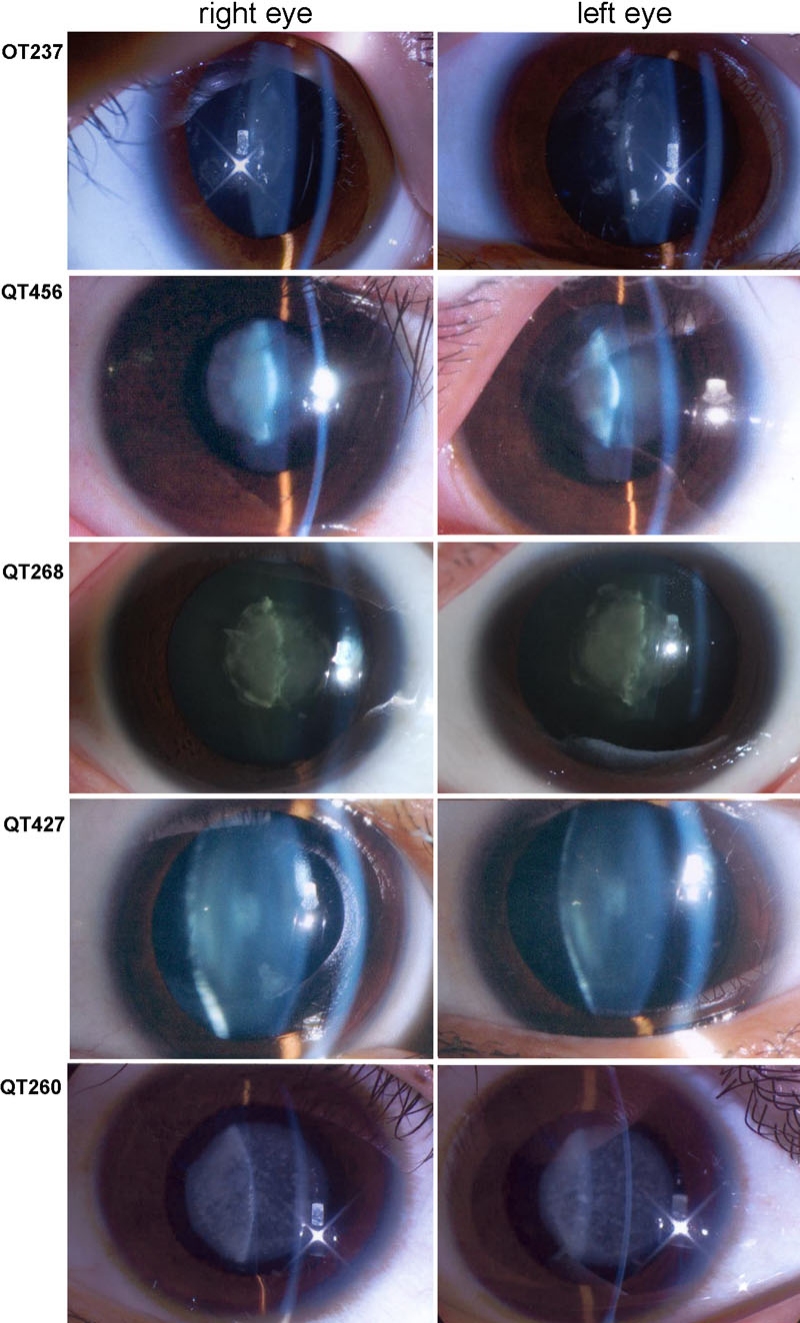
Lens photos showing cataract phenotypes in probands with identified mutations. Family number of each proband was listed in left column. The proband from family QT237 with the c.292G>A mutation in *CRYAA* had bilateral lamellar and punctate cataract. The proband from QT456 with the c.272–274delGAG mutation in *CRYBA1* had bilateral nuclear cataract. The proband from QT268 with the c.106G>C mutation in *CRYGD* had bilateral nuclear cataract. The proband from QT427 with the c.77 A>G in *CRYGS* showed bilateral coppock cataract. The proband from QT260 with the c.1143–1165del23 mutation in *GJA3* had bilateral punctate nuclear cataract.

The clinical data of the available patients with mutations were listed in Table 3 and cataract phenotypes for some patients were shown in Figure 4.

**Table 3:** The clinical information of the patients with congenital cataracts and identified mutations.

**ID**	**Gene**	**Mutation**	**Gender**	**Age (yrs) at**		**Inheritance**	**Visual acuity (right;left)**	**Cataract types**
** **	** **	** **	** **	**exam**	**onset**	** **	** **	** **
QT237	*CRYAA*	c.292G>A	male	10	7	AD	0.2; 0.5	lamellar, punctate
QT237 II:1	*CRYAA*	c.292G>A	female	N/A	N/A	AD	0.6; 0.7	lamellar, Y-suture
QT261	*CRYAA*	c.350_352delGCT	male	5	at birth	AD	N/A; 0.2	N/A
QT192	*CRYAB*	c.205 C>T	male	N/A	N/A	AD	N/A	N/A
QT286	*CRYBA1*	c.215+1G>A	male	6	at birth	AD	0.3; 0.1	lamellar
QT456	*CRYBA1*	c.272_274delGAG	male	19	at birth	AD	0.1; 0.1	nuclear
QT174	*CRYBA1*	c.272_274delGAG	female	49	at birth	AD	FC; FC	nuclear
QT268	*CRYGD*	c.106G>C	male	40	at birth	AD	0.3; 0.2	nuclear
QT427	*CRYGS*	c.77 A>G	female	27	at birth	sporadic	N/A	coppock
QT206	*GJA3*	c.176 C>T	female	26	at birth	AD	0.6; 0.5	N/A
QT260	*GJA3*	c.1143_1165del23	female	17	at birth	AD	0.4; 0.2	punctate nuclear

FC: Finger counting. N/A: not available. AD: autosomal dominant.

## Discussion

In this study, nine heterozygous mutations in the 12 genes encoding crystallins and connexins were identified in 10 out of 25 Chinese families (40%) with congenital cataract. Eight of the 25 families (32%) had mutations in crystallin genes and two of them (8%) had mutations in connexin genes.

For the congenital cataract families with identified mutations reported before, about three-fourths of the families had mutations in the 10 crystallin and 2 connexin genes, which was mostly based on studies of individual gene [3]. However, this mutation proportion may not reflect the actual frequency of these genes in congenital cataract, since the genes encoding crystallins and connexins might be more frequently analyzed. In fact, varied frequencies of mutation detection, mostly lower, were reported in several studies involving analysis of multiple genes. Devi et al. found crystallin mutations in 16.6% (10 of 60) Indian families by analyzing the 10 crystallin genes [10]. Burdon et al. [63] detected crystallin mutations in 5.3% (2/38) Australian families by analyzing 7 crystallin genes. Hansen et al. [36] detected crystallin and connexin mutations in 35.7% (10/28) and 21.4% (6/28) Danish families, respectively. Kumar et al. [40] detected mutations in 20% (6/30) Indian families by analyzing 4 of the 12 genes. Wang et al. [29] identified mutations in 15% (3/20) Chinese families by analyzing 10 of the 12 genes. None of these studies performed analysis of all the 12 genes in the same set of families. In this study, we detected mutations in 40% families by analyzing all of the 12 genes. Overall, the frequencies of mutations in the 12 genes varied significantly in different ethnic populations but were more likely to be less than three fourth of families with congenital cataracts. The over-representation of mutations in these 12 genes is more likely due to more frequent studies on these genes.

Although several mutations in the 12 genes have been identified so far, we still identified five novel mutations in the Chinese families with congenital cataracts. Our results expand the mutation spectrum of these genes in Chinese population. The families without identified mutations will be good candidates for future study in screening for additional causative genes.

## Appendix 1

Primers used to amplify the coding and adjacent regions of the 12 genes. To access the data, click or select the words “Appendix 1.” This will initiate the download of a Microsoft Excel (.xml) file that contains the data.

## References

[1] Wilson ME, Pandey SK, Thakur J (2003). Paediatric cataract blindness in the developing world: surgical techniques and intraocular lenses in the new millennium.. Br J Ophthalmol.

[2] Reddy MA, Francis PJ, Berry V, Bhattacharya SS, Moore AT (2004). Molecular genetic basis of inherited cataract and associated phenotypes.. Surv Ophthalmol.

[3] Hejtmancik JF (2008). Congenital cataracts and their molecular genetics.. Semin Cell Dev Biol.

[4] Shiels A, Bennett TM, Hejtmancik JF (2010). Cat-Map: putting cataract on the map.. Mol Vis.

[5] Litt M, Kramer P, LaMorticella DM, Murphey W, Lovrien EW, Weleber RG (1998). Autosomal dominant congenital cataract associated with a missense mutation in the human alpha crystallin gene CRYAA.. Hum Mol Genet.

[6] Pras E, Frydman M, Levy-Nissenbaum E, Bakhan T, Raz J, Assia EI, Goldman B, Pras E (2000). A nonsense mutation (W9X) in CRYAA causes autosomal recessive cataract in an inbred Jewish Persian family.. Invest Ophthalmol Vis Sci.

[7] Santhiya ST, Soker T, Klopp N, Illig T, Prakash MV, Selvaraj B, Gopinath PM, Graw J (2006). Identification of a novel, putative cataract-causing allele in CRYAA (G98R) in an Indian family.. Mol Vis.

[8] Vanita V, Singh JR, Hejtmancik JF, Nuernberg P, Hennies HC, Singh D, Sperling K (2006). A novel fan-shaped cataract-microcornea syndrome caused by a mutation of CRYAA in an Indian family.. Mol Vis.

[9] Graw J, Klopp N, Illig T, Preising MN, Lorenz B (2006). Congenital cataract and macular hypoplasia in humans associated with a de novo mutation in CRYAA and compound heterozygous mutations in P.. Graefes Arch Clin Exp Ophthalmol.

[10] Devi RR, Yao W, Vijayalakshmi P, Sergeev YV, Sundaresan P, Hejtmancik JF (2008). Crystallin gene mutations in Indian families with inherited pediatric cataract.. Mol Vis.

[11] Gu F, Luo W, Li X, Wang Z, Lu S, Zhang M, Zhao B, Zhu S, Feng S, Yan YB, Huang S, Ma X (2008). A novel mutation in AlphaA-crystallin (CRYAA) caused autosomal dominant congenital cataract in a large Chinese family.. Hum Mutat.

[12] Santana A, Waiswol M, Arcieri ES, Cabral de Vasconcellos JP, Barbosa de Melo M (2009). Mutation analysis of CRYAA, CRYGC, and CRYGD associated with autosomal dominant congenital cataract in Brazilian families.. Mol Vis.

[13] Berry V, Francis P, Reddy MA, Collyer D, Vithana E, MacKay I, Dawson G, Carey AH, Moore A, Bhattacharya SS, Quinlan RA (2001). Alpha-B crystallin gene (CRYAB) mutation causes dominant congenital posterior polar cataract in humans.. Am J Hum Genet.

[14] Liu Y, Zhang X, Luo L, Wu M, Zeng R, Cheng G, Hu B, Liu B, Liang JJ, Shang F (2006). A novel alphaB-crystallin mutation associated with autosomal dominant congenital lamellar cataract.. Invest Ophthalmol Vis Sci.

[15] Safieh LA, Khan AO, Alkuraya FS (2009). Identification of a novel CRYAB mutation associated with autosomal recessive juvenile cataract in a Saudi family.. Mol Vis.

[16] Kannabiran C, Rogan PK, Olmos L, Basti S, Rao GN, Kaiser-Kupfer M, Hejtmancik JF (1998). Autosomal dominant zonular cataract with sutural opacities is associated with a splice mutation in the betaA3/A1-crystallin gene.. Mol Vis.

[17] Bateman JB, Geyer DD, Flodman P, Johannes M, Sikela J, Walter N, Moreira AT, Clancy K, Spence MA (2000). A new betaA1-crystallin splice junction mutation in autosomal dominant cataract.. Invest Ophthalmol Vis Sci.

[18] Reddy MA, Bateman OA, Chakarova C, Ferris J, Berry V, Lomas E, Sarra R, Smith MA, Moore AT, Bhattacharya SS, Slingsby C (2004). Characterization of the G91del CRYBA1/3-crystallin protein: a cause of human inherited cataract.. Hum Mol Genet.

[19] Gu Z, Ji B, Wan C, He G, Zhang J, Zhang M, Feng G, He L, Gao L (2010). A splice site mutation in CRYBA1/A3 causing autosomal dominant posterior polar cataract in a Chinese pedigree.. Mol Vis.

[20] Zhu Y, Shentu X, Wang W, Li J, Jin C, Yao K (2010). A Chinese family with progressive childhood cataracts and IVS3+1G>A CRYBA3/A1 mutations.. Mol Vis.

[21] Qi Y, Jia H, Huang S, Lin H, Gu J, Su H, Zhang T, Gao Y, Qu L, Li D, Li Y (2004). A deletion mutation in the betaA1/A3 crystallin gene (CRYBA1/A3) is associated with autosomal dominant congenital nuclear cataract in a Chinese family.. Hum Genet.

[22] Ferrini W, Schorderet DF, Othenin-Girard P, Uffer S, Heon E, Munier FL (2004). CRYBA3/A1 gene mutation associated with suture-sparing autosomal dominant congenital nuclear cataract: a novel phenotype.. Invest Ophthalmol Vis Sci.

[23] Lu S, Zhao C, Jiao H, Kere J, Tang X, Zhao F, Zhang X, Zhao K, Larsson C (2007). Two Chinese families with pulverulent congenital cataracts and deltaG91 CRYBA1 mutations.. Mol Vis.

[24] Billingsley G, Santhiya ST, Paterson AD, Ogata K, Wodak S, Hosseini SM, Manisastry SM, Vijayalakshmi P, Gopinath PM, Graw J, Heon E (2006). CRYBA4, a novel human cataract gene, is also involved in microphthalmia.. Am J Hum Genet.

[25] Zhou G, Zhou N, Hu S, Zhao L, Zhang C, Qi Y (2010). A missense mutation in CRYBA4 associated with congenital cataract and microcornea.. Mol Vis.

[26] Mackay DS, Boskovska OB, Knopf HL, Lampi KJ, Shiels A (2002). A nonsense mutation in CRYBB1 associated with autosomal dominant cataract linked to human chromosome 22q.. Am J Hum Genet.

[27] Cohen D, Bar-Yosef U, Levy J, Gradstein L, Belfair N, Ofir R, Joshua S, Lifshitz T, Carmi R, Birk OS (2007). Homozygous CRYBB1 deletion mutation underlies autosomal recessive congenital cataract.. Invest Ophthalmol Vis Sci.

[28] Meyer E, Rahman F, Owens J, Pasha S, Morgan NV, Trembath RC, Stone EM, Moore AT, Maher ER (2009). Initiation codon mutation in betaB1-crystallin (CRYBB1) associated with autosomal recessive nuclear pulverulent cataract.. Mol Vis.

[29] Wang KJ, Wang BB, Zhang F, Zhao Y, Ma X, Zhu SQ (2011). Novel beta-crystallin gene mutations in Chinese families with nuclear cataracts.. Arch Ophthalmol.

[30] Litt M, Carrero-Valenzuela R, LaMorticella DM, Schultz DW, Mitchell TN, Kramer P, Maumenee IH (1997). Autosomal dominant cerulean cataract is associated with a chain termination mutation in the human beta-crystallin gene CRYBB2.. Hum Mol Genet.

[31] Bateman JB, von-Bischhoffshaunsen FR, Richter L, Flodman P, Burch D, Spence MA (2007). Gene conversion mutation in crystallin, beta-B2 (CRYBB2) in a Chilean family with autosomal dominant cataract.. Ophthalmology.

[32] Li FF, Zhu SQ, Wang SZ, Gao C, Huang SZ, Zhang M, Ma X (2008). Nonsense mutation in the CRYBB2 gene causing autosomal dominant progressive polymorphic congenital coronary cataracts.. Mol Vis.

[33] Wang L, Lin H, Gu J, Su H, Huang S, Qi Y (2009). Autosomal-dominant cerulean cataract in a chinese family associated with gene conversion mutation in beta-B2-crystallin.. Ophthalmic Res.

[34] Santhiya ST, Kumar GS, Sudhakar P, Gupta N, Klopp N, Illig T, Soker T, Groth M, Platzer M, Gopinath PM, Graw J (2010). Molecular analysis of cataract families in India: new mutations in the CRYBB2 and GJA3 genes and rare polymorphisms.. Mol Vis.

[35] Riazuddin SA, Yasmeen A, Yao W, Sergeev YV, Zhang Q, Zulfiqar F, Riaz A, Riazuddin S, Hejtmancik JF (2005). Mutations in betaB3-crystallin associated with autosomal recessive cataract in two Pakistani families.. Invest Ophthalmol Vis Sci.

[36] Hansen L, Mikkelsen A, Nurnberg P, Nurnberg G, Anjum I, Eiberg H, Rosenberg T (2009). Comprehensive mutational screening in a cohort of Danish families with hereditary congenital cataract.. Invest Ophthalmol Vis Sci.

[37] Héon E, Priston M, Schorderet DF, Billingsley GD, Girard PO, Lubsen N, Munier FL (1999). The gamma-crystallins and human cataracts: a puzzle made clearer.. Am J Hum Genet.

[38] Yao K, Jin C, Zhu N, Wang W, Wu R, Jiang J, Shentu X (2008). A nonsense mutation in CRYGC associated with autosomal dominant congenital nuclear cataract in a Chinese family.. Mol Vis.

[39] Zhang L, Fu S, Ou Y, Zhao T, Su Y, Liu P (2009). A novel nonsense mutation in CRYGC is associated with autosomal dominant congenital nuclear cataracts and microcornea.. Mol Vis.

[40] Kumar M, Agarwal T, Khokhar S, Kumar M, Kaur P, Roy TS, Dada R (2011). Mutation screening and genotype phenotype correlation of alpha-crystallin, gamma-crystallin and GJA8 gene in congenital cataract.. Mol Vis.

[41] Kmoch S, Brynda J, Asfaw B, Bezouska K, Novak P, Rezacova P, Ondrova L, Filipec M, Sedlacek J, Elleder M (2000). Link between a novel human gammaD-crystallin allele and a unique cataract phenotype explained by protein crystallography.. Hum Mol Genet.

[42] Zenteno JC, Morales ME, Moran-Barroso V, Sanchez-Navarro A (2005). CRYGD gene analysis in a family with autosomal dominant congenital cataract: evidence for molecular homogeneity and intrafamilial clinical heterogeneity in aculeiform cataract.. Mol Vis.

[43] Hansen L, Yao W, Eiberg H, Kjaer KW, Baggesen K, Hejtmancik JF, Rosenberg T (2007). Genetic heterogeneity in microcornea-cataract: five novel mutations in CRYAA, CRYGD, and GJA8.. Invest Ophthalmol Vis Sci.

[44] Sun H, Ma Z, Li Y, Liu B, Li Z, Ding X, Gao Y, Ma W, Tang X, Li X, Shen Y (2005). Gamma-S crystallin gene (CRYGS) mutation causes dominant progressive cortical cataract in humans.. J Med Genet.

[45] Vanita V, Singh JR, Singh D, Varon R, Sperling K (2009). Novel mutation in the gamma-S crystallin gene causing autosomal dominant cataract.. Mol Vis.

[46] Mackay D, Ionides A, Kibar Z, Rouleau G, Berry V, Moore A, Shiels A, Bhattacharya S (1999). Connexin46 mutations in autosomal dominant congenital cataract.. Am J Hum Genet.

[47] Devi RR, Reena C, Vijayalakshmi P (2005). Novel mutations in GJA3 associated with autosomal dominant congenital cataract in the Indian population.. Mol Vis.

[48] Addison PK, Berry V, Holden KR, Espinal D, Rivera B, Su H, Srivastava AK, Bhattacharya SS (2006). A novel mutation in the connexin 46 gene (GJA3) causes autosomal dominant zonular pulverulent cataract in a Hispanic family.. Mol Vis.

[49] Guleria K, Sperling K, Singh D, Varon R, Singh JR, Vanita V (2007). A novel mutation in the connexin 46 (GJA3) gene associated with autosomal dominant congenital cataract in an Indian family.. Mol Vis.

[50] Bennett TM, Mackay DS, Knopf HL, Shiels A (2004). A novel missense mutation in the gene for gap-junction protein alpha3 (GJA3) associated with autosomal dominant “nuclear punctate” cataracts linked to chromosome 13q.. Mol Vis.

[51] Shiels A, Mackay D, Ionides A, Berry V, Moore A, Bhattacharya S (1998). A missense mutation in the human connexin50 gene (GJA8) underlies autosomal dominant “zonular pulverulent” cataract, on chromosome 1q.. Am J Hum Genet.

[52] Vanita V, Hennies HC, Singh D, Nurnberg P, Sperling K, Singh JR (2006). A novel mutation in GJA8 associated with autosomal dominant congenital cataract in a family of Indian origin.. Mol Vis.

[53] Arora A, Minogue PJ, Liu X, Reddy MA, Ainsworth JR, Bhattacharya SS, Webster AR, Hunt DM, Ebihara L, Moore AT, Beyer EC, Berthoud VM (2006). A novel GJA8 mutation is associated with autosomal dominant lamellar pulverulent cataract: further evidence for gap junction dysfunction in human cataract.. J Med Genet.

[54] Ponnam SP, Ramesha K, Tejwani S, Ramamurthy B, Kannabiran C (2007). Mutation of the gap junction protein alpha 8 (GJA8) gene causes autosomal recessive cataract.. J Med Genet.

[55] Schmidt W, Klopp N, Illig T, Graw J (2008). A novel GJA8 mutation causing a recessive triangular cataract.. Mol Vis.

[56] Yan M, Xiong C, Ye SQ, Chen Y, Ke M, Zheng F, Zhou X (2008). A novel connexin 50 (GJA8) mutation in a Chinese family with a dominant congenital pulverulent nuclear cataract.. Mol Vis.

[57] Zhou Z, Hu S, Wang B, Zhou N, Zhou S, Ma X, Qi Y (2010). Mutation analysis of congenital cataract in a Chinese family identified a novel missense mutation in the connexin 46 gene (GJA3).. Mol Vis.

[58] Jiang H, Jin Y, Bu L, Zhang W, Liu J, Cui B, Kong X, Hu L (2003). A novel mutation in GJA3 (connexin46) for autosomal dominant congenital nuclear pulverulent cataract.. Mol Vis.

[59] Zhang X, Li S, Xiao X, Jia X, Wang P, Shen H, Guo X, Zhang Q (2009). Mutational screening of 10 genes in Chinese patients with microphthalmia and/or coloboma.. Mol Vis.

[60] Hicks S, Wheeler DA, Plon SE, Kimmel M (2011). Prediction of missense mutation functionality depends on both the algorithm and sequence alignment employed.. Hum Mutat.

[61] Zou M, Baitei EY, Alzahrani AS, Parhar RS, Al-Mohanna FA, Meyer BF, Shi Y (2011). Mutation prediction by PolyPhen or functional assay, a detailed comparison of CYP27B1 missense mutations.. Endocrine.

[62] Ng PC, Henikoff S (2003). SIFT: Predicting amino acid changes that affect protein function.. Nucleic Acids Res.

[63] Burdon KP, Wirth MG, Mackey DA, Russell-Eggitt IM, Craig JE, Elder JE, Dickinson JL, Sale MM (2004). Investigation of crystallin genes in familial cataract, and report of two disease associated mutations.. Br J Ophthalmol.

